# Oral Health and Oral Health-Related Quality of Life in Patients Undergoing Inpatient Treatment for Heroin-Related Opioid Use Disorder

**DOI:** 10.3390/medicina62081458

**Published:** 2026-07-28

**Authors:** Nina Dimitrijevic Jovanovic, Maja Milosevic Markovic, Jovana Kuzmanovic-Pficer, Vesna Bjegovic-Mikanovic, Srdjan Milovanovic, Ivan Dozic, Sanja Medenica, Natasa Pejcic, Neda Perunovic, Dejan Calasan, Milica Jaksic Karisik, Svetlana Jovanovic

**Affiliations:** 1 School of Dental Medicine, University of Belgrade, 11000 Belgrade, Serbia; nina.dimitrijevic.jovanovic@stomf.bg.ac.rs (N.D.J.); jovana.kuzmanovic@stomf.bg.ac.rs (J.K.-P.); ivan.dozic@stomf.bg.ac.rs (I.D.); natasa.pejcic@stomf.bg.ac.rs (N.P.); neda.perunovic@stomf.bg.ac.rs (N.P.); dejan.calasan@stomf.bg.ac.rs (D.C.); milica.jaksic@stomf.bg.ac.rs (M.J.K.); svetlana.jovanovic@stomf.bg.ac.rs (S.J.); 2 Institute of Social Medicine, Faculty of Medicine, University of Belgrade, 11000 Belgrade, Serbia; vesna.bjegovic-mikanovic@med.bg.ac.rs; 3 Clinic for Psychiatry, University Clinical Center of Serbia, Faculty of Medicine, University of Belgrade, 11000 Belgrade, Serbia; srdjan.milovanovic@med.bg.ac.rs; 4 Institute of Public Health of Montenegro, 81000 Podgorica, Montenegro; sanja.medenica@ijzcg.me

**Keywords:** heroin-related opioid use disorder, oral health-related quality of life, oral health status, dental caries, oral hygiene

## Abstract

*Background and Objectives:* Individuals with heroin-related opioid use disorder are at high risk of poor oral health and impaired oral health-related quality of life (OHRQoL). However, data on the determinants of OHRQoL in this population remain limited. This study aimed to assess oral health status and OHRQoL and to identify key predictors of OHRQoL among hospitalized patients with heroin-related opioid use disorder. *Materials and Methods*: A cross-sectional study was conducted among 97 hospitalized patients with heroin-related opioid use disorder. Data were collected through structured interviews and clinical oral examinations, including the Decayed, Missing, and Filled Teeth (DMFT) index and the Simplified Oral Hygiene Index (OHI-S). OHRQoL was assessed using the Oral Health Impact Profile-14 (OHIP-14). Spearman correlation and multiple linear regression analyses were performed to examine associations and identify predictors of OHRQoL. *Results*: Participants had poor oral health, with high DMFT (19.1 ± 6.1) and OHI-S (2.0 ± 1.2) scores. OHRQoL was markedly impaired, with a mean OHIP-14 score of 29.6 ± 7.0, particularly in the psychological and social domains. OHIP-14 scores were strongly correlated with DMFT (r = 0.754, *p* < 0.001) and OHI-S (r = 0.552, *p* < 0.001). Longer duration of heroin use was associated with higher DMFT scores, poorer oral hygiene, and greater OHRQoL impairment. Multiple linear regression analysis identified unstable housing/homelessness (β = 0.166, *p* = 0.012), DMFT (β = 0.624, *p* < 0.001), and OHI-S (β = 0.272, *p* = 0.007) as significant predictors of poorer OHRQoL. *Conclusions*: Individuals with heroin-related opioid use disorder experienced substantial oral health burden and impaired OHRQoL, strongly associated with caries experience, poor oral hygiene, and housing instability. These findings highlight the need to integrate prevention-oriented dental care into addiction treatment services and to address the broader social determinants of oral health.

## 1. Introduction

Illicit drug use represents a major public health challenge, with widespread consequences at both individual and societal levels. According to a recent report, the global number of drug users reached 292 million in 2022, reflecting a 20% increase over the past decade [1]. Heroin remains one of the most widely used illicit opioids and contributes substantially to the overall burden associated with substance use [2]. In Serbia, heroin is among the most commonly injected drugs, with a lifetime prevalence of 0.4% among individuals aged 15–64 years [3].

Substance use disorders are associated with significant impairments in physical, psychological, and social functioning [4]. Individuals with heroin use disorder often experience a range of oral health complications, including dental caries, tooth loss, periodontal disease, xerostomia, bruxism, tooth erosion, and temporomandibular disorders [5]. These conditions may arise from both the direct effects of drug use on the oral cavity and indirect factors such as poor hygiene, inadequate nutrition, and limited access to dental care. Untreated oral conditions may result in pain, infection, tooth loss, reduced masticatory function, and diminished overall well-being [6,7].

Multiple healthcare interventions are available for individuals with opioid use disorder, including pharmacotherapies such as methadone, which is a well-established, evidence-based, and cost-effective treatment associated with improved clinical outcomes and reduced morbidity and mortality [8]. In Serbia, methadone therapy is routinely implemented in clinical settings as part of standard care for patients with opioid use disorder [3].

Oral health-related quality of life (OHRQoL) is a multidimensional concept reflecting the impact of oral health on daily functioning and overall well-being [9]. It encompasses functional, psychological, and social dimensions of health. Given the high burden of oral disease among individuals with heroin use disorder, identifying factors that influence OHRQoL in this population is essential for understanding unmet needs and developing targeted interventions [10].

The Oral Health Impact Profile (OHIP-14) is one of the most widely used instruments for assessing OHRQoL [11]. It is a concise, psychometrically robust tool designed to capture the functional and psychosocial consequences of oral disorders [12] and has been validated across diverse populations, including people who use drugs [13,14,15], as well as for use in the Serbian linguistic and cultural context [16].

Despite increasing recognition of oral health problems among individuals with substance use disorders, evidence on the determinants of OHRQoL among patients with heroin-related opioid use disorder remains limited. Therefore, this study aimed to assess oral health status and OHRQoL in this population and to identify clinical, behavioral, and socio-demographic predictors of OHRQoL.

## 2. Materials and Methods

### 2.1. Study Sample

A cross-sectional study was conducted from August 2022 to December 2023 and included 97 hospitalized patients with heroin-related opioid use disorder (ICD-10: F11.2, dependence syndrome) at the Special Hospital for Addiction Diseases in Belgrade, Serbia, within the public healthcare system, where participants received methadone as part of a supervised inpatient detoxification program. Written informed consent was obtained from all participants after the purpose and procedures of the study had been fully explained to them. The study was approved by the Ethics Committee of the School of Dental Medicine, University of Belgrade (No. 36/20), and by the Ethics Committee of the Special Hospital for Addiction Diseases, Belgrade (No. 11772). The study was conducted in accordance with the Declaration of Helsinki.

Eligible participants were required to be at least 18 years old; have no clinically diagnosed systemic diseases other than documented HCV and/or HIV antibody positivity; be proficient in Serbian; have no cognitive impairments that would prevent questionnaire completion, as assessed by a psychiatrist; and express willingness to undergo a detailed dental exam and to complete an OHRQoL assessment. Patients who had been receiving methadone treatment for more than one month were not eligible. Participants were recruited consecutively in the order of presentation during the study period, and all eligible patients who met the inclusion criteria were invited to participate.

The sample size was calculated for a single-sample estimate of the mean, assuming a standard deviation of 9.48 [14] and a desired precision (margin of error) of 2 units at a 95% confidence level. Based on these parameters, the minimum required sample size was 86 participants. A total of 97 participants were included in the study, providing greater precision and a narrower confidence interval than initially planned.

### 2.2. Data Collection

Data were collected from all participants through intraoral clinical examinations, structured interviews, and hospital records.

The oral clinical examinations included assessment of the DMFT index (Decayed, Missing, and Filled Teeth), as well as its components DT (Decayed Teeth), MT (Missing Teeth), and FT (Filled Teeth), and evaluation of oral hygiene status using the Simplified Oral Hygiene Index (OHI-S), which consists of the Debris Index (DI) and Calculus Index (CI) [17].

Clinical examination was conducted by two experienced dentists according to WHO requirements [18]. The examiners (N.D.J. and M.M.M.) underwent training and calibration on 25 participants not involved in the study, with weighted Kappa for intra-rater reliability 0.881–0.919 and for inter-rater reliability 0.821–0.861 (95% CI 0.708–0.992; *p* < 0.001). Each participant was examined while sitting in a dental chair under artificial light, using pre-packaged sterilized instruments (single-use mirror and periodontal probe). The dental examination was performed during the inpatient detoxification period, approximately two weeks after the last reported heroin use, once the acute phase of opioid withdrawal had subsided and the patients were clinically stable.

After the dental examination, each participant underwent a structured interview lasting 10–15 min. The interview included questions about socio-demographic information (age, sex, marital status, education level, employment status, accommodation, and self-perceived economic status); risk behaviors (smoking status, smoking duration, number of cigarettes smoked per day, and alcohol consumption, defined as self-reported intake exceeding moderate drinking limits, namely, >2 standard drinks per day for men and >1 standard drink per day for women [19]); and oral health characteristics (tooth brushing habits, last dental visit, and self-assessed oral health). Xerostomia was recorded as self-reported dry mouth, while bruxism was assessed based on self-reported tooth grinding or clenching and was therefore classified as possible bruxism according to the international consensus grading system [20].

Data regarding the primary disease in the study group were obtained from hospital records. These data included the following: drug abuse history (duration and patterns of heroin abuse), types of other drugs used, duration of methadone therapy during hospitalization (in days), and hepatitis C virus (HCV) and HIV antibody positivity. For descriptive purposes, duration of heroin use was dichotomized at five years to facilitate group comparison and interpretation.

OHRQoL was measured using the Serbian version of the Oral Health Impact Profile (OHIP-14) [12,16]. The OHIP-14 includes 14 items and covers seven domains: functional limitation, physical pain, psychological discomfort, physical disability, psychological disability, social disability, and handicap, with two questions dedicated to each domain. The response options are scored on a five-point Likert scale (0 = Never, 1 = Hardly ever, 2 = Occasionally, 3 = Fairly often, and 4 = Very often). A higher score is generally indicative of worse oral health. Based on previous studies, we used three different OHIP-14 summary measurements to assess OHRQoL [13,21].

“Prevalence” was defined as the proportion of participants who reported “fairly often” or “very often” on at least one OHIP-14 item, indicating the presence of an impact [22]. The “severity” was assessed based on the total score (0–56 points), with a higher score indicating a greater perceived negative impact by the participant [22]. “Extent” was the number of items (0–14) reported at least “very often” and “fairly often” [23]. Cronbach’s alpha coefficients were ≧ 0.70. The OHIP-14 was administered in an interview format by two trained researchers (N.D.J. and M.M.M.), with a duration of 10–15 min.

### 2.3. Data Analyses

Data were analyzed using SPSS version 22 (SPSS Inc., Chicago, IL, USA). Continuous variables are presented as means ± standard deviations (SDs), while categorical variables are shown as frequencies and percentages. The chi-square test was used for categorical data. Normality was assessed using the Kolmogorov–Smirnov test. Depending on distribution, either the independent *t*-test or the Mann–Whitney U test was applied to compare groups. Spearman correlation was used to explore associations with OHIP-14 scores. Univariate and multiple linear regression analyses were conducted to identify predictors of OHRQoL. Variables significant in univariable analyses were entered into the multivariable regression model.

Prior to interpreting the multiple linear regression model, the assumptions of linear regression were assessed. Normality of residuals was evaluated using histograms and normal P–P plots of standardized residuals. Linearity and homoscedasticity were assessed by visual inspection of scatterplots of standardized residuals against standardized predicted values. Multicollinearity was evaluated using tolerance and variance inflation factor (VIF) values. Statistical significance was set at *p* ≤ 0.05.

## 3. Results

### 3.1. Participant Characteristics

The sample included 97 hospitalized patients with heroin-related opioid use disorder, with a mean age of 26.2 ± 3.7 years. Socio-demographic characteristics and oral hygiene practices are summarized in Table 1.

Participants exhibited pronounced socioeconomic disadvantage, being predominantly single, with secondary-level education and high unemployment rates; more than one-third experienced unstable housing or homelessness, and a comparable proportion reported poor or very poor self-perceived economic status. Participants were predominantly smokers, with the majority reporting consumption of more than 20 cigarettes per day, while over one-third reported alcohol use.

### 3.2. Oral Health Status

Regarding oral health behaviors, 13 participants (13.4%) reported not brushing their teeth, 43 (44.3%) brushed once daily, and 41 (42.3%) brushed two or more times per day. Most participants, 75 (77.3%), reported that their last dental visit had occurred more than one year earlier, whereas 22 (22.7%) had visited a dentist within the previous year. Self-assessed oral health was rated as bad or very bad by 57 participants (58.8%), moderate by 33 (34.0%), and good or very good by 7 participants (7.2%).

Possible bruxism and self-reported xerostomia were recorded in 41 (42.3%) and 27 (27.8%) participants, respectively. The DMFT index was high (19.1 ± 6.1), with decayed teeth (DT) being the most prevalent component (11.5 ± 4.9), followed by missing teeth (MT) (5.0 ± 3.5) and filled teeth (FT) (2.6 ± 2.3). The mean OHI-S score was 2.0 ± 1.2, with a greater contribution from the Debris Index (1.6 ± 1.0) compared to the Calculus Index (0.4 ± 0.3).

### 3.3. Substance Use Patterns and Comorbid Infections

In the study sample, 63.9% of participants reported heroin use for more than 5 years, and the mean duration of heroin use was 117.3 ± 49.3 months. Injection was the most common route of administration (70.1%), followed by snorting (17.5%), inhalation (8.3%), and combined methods (4.1%). Polydrug use was highly prevalent, with 72.2% reporting the use of additional substances, including sedatives (82%), marijuana (80%), amphetamines (70%), cocaine (60%), and hallucinogens (48%). The mean duration of methadone therapy during hospitalization was 15 ± 10 days. More than half of the participants (50.5%) tested positive for HCV, and one individual (1.0%) was HIV-positive.

### 3.4. Quality of Life

In the total sample, the mean OHIP-14 severity score was 29.6 ± 7.0. The highest mean domain scores were observed for handicap (5.6 ± 1.7), psychological discomfort (5.3 ± 1.6), psychological disability (5.1 ± 1.8), and social disability (4.6 ± 2.0) (Table 2). The prevalence of OHRQoL impact was high, with 95 participants (97.9%) reporting at least one OHIP-14 impact as “fairly often” or “very often”. The highest prevalence of impact was observed for psychological discomfort (86 participants, 88.7%), handicap (85 participants, 87.6%), psychological disability (72 participants, 74.2%), and social disability (66 participants, 68.0%). The mean extent of impact was 3.0 ± 1.9.

The relationship between duration of heroin use and oral health indicators is presented in Table 2. In the exploratory stratified analysis using a five-year cut-off for descriptive purposes, no clear differences were observed in tooth brushing frequency or dental visits between groups. However, individuals with more than five years of heroin use more frequently reported poor self-assessed oral health (*p* < 0.05) and showed a higher prevalence of possible bruxism. They also had significantly higher DMFT scores, particularly in decayed and missing components, as well as elevated Debris Index, Calculus Index, and OHI-S values (all *p* < 0.05), indicating poorer oral hygiene. Additionally, longer duration of heroin use was associated with higher OHIP-14 total scores, with significant differences observed in the domains of physical pain, psychological discomfort, and physical disability (*p* < 0.05).

Correlation analysis revealed significant associations between OHIP-14 severity scores and several clinical and subjective variables (Table 3). Higher OHIP-14 scores were strongly associated with higher DMFT values (r = 0.754, *p* < 0.001), particularly with the decayed and missing components of the index. Poorer oral hygiene was also significantly associated with higher OHIP-14 scores, as indicated by correlations with OHI-S (r = 0.552, *p* < 0.001), Debris Index (r = 0.575, *p* < 0.001), and Calculus Index (r = 0.301, *p* = 0.003). A significant negative correlation was observed with self-assessed oral health (r = −0.267, *p* = 0.008), while no association was found with self-perceived economic status (*p* = 0.988). Duration of heroin use was also positively associated with OHIP-14 severity (r = 0.384, *p* < 0.001).

The diagnostic check showed that the assumptions of multiple linear regression were met. Standardized residuals ranged from −2.59 to 2.53, indicating no extreme outliers. Looking at histograms and normal P–P plots showed the residuals were roughly normally distributed, and scatterplots of standardized residuals against predicted values showed no major problems with linearity or equal spread of errors. There was no sign of multicollinearity, with tolerance values between 0.37 and 0.87 and VIF values between 1.15 and 2.71.

Multiple linear regression analysis (adjusted R^2^ = 0.685; F = 27.709; *p* < 0.001) identified three variables significantly associated with poorer OHRQoL: accommodation status (β = 0.166, *p* = 0.012), with higher OHIP-14 scores observed among participants living in unstable housing conditions or experiencing homelessness; DMFT index (β = 0.624, *p* < 0.001), indicating that greater caries experience was associated with poorer OHRQoL; and OHI-S index (β = 0.272, *p* = 0.007), indicating that poorer oral hygiene was associated with higher perceived oral health impacts (Table 4).

## 4. Discussion

This study assessed oral health status and oral health-related quality of life (OHRQoL) among hospitalized patients with heroin-related opioid use disorder and found a strong association between poorer oral health status and impaired OHRQoL in this population.

Participants in our sample appeared to experience substantial social and economic vulnerability, consistent with previous research [4,6,21,24,25]. These results align with the broader literature highlighting structural inequalities, social exclusion, and economic marginalization commonly experienced by individuals with substance use disorders [14,26]. Behavioral risk factors, including long-term tobacco use and frequent alcohol consumption, were also highly prevalent in this group [27,28,29]. These factors, together with suboptimal oral hygiene practices and limited utilization of dental services, likely contribute to the poor oral health outcomes observed in this population, in line with previous studies [21,27,30,31,32,33]. It should also be noted that, because all participants were under treatment and hospitalized, their oral hygiene habits may not fully correspond to those of non-hospitalized heroin users.

In this population, the presence of bruxism and xerostomia may indicate a notable burden of oral parafunctional activity and salivary dysfunction. This finding may partly reflect the overlapping effects of heroin-related opioid use disorder and supervised methadone treatment. It is also consistent with previous studies linking bruxism to neuromuscular effects and psychosocial stress and xerostomia to substance use and medication-related side effects, including methadone therapy [27,34,35]. Although less prevalent than bruxism, xerostomia remains clinically relevant due to its contribution to impaired oral health and increased risk of dental disease [27].

People with substance use disorders are known to experience a substantial burden of dental disease, including caries, tooth loss, and restorative needs [36,37,38]. Pharmacotherapy used in addiction treatment may further contribute to caries risk through its association with increased preference for sweet foods and beverages, which can compound the effect of frequent sugar exposure in this population [39]. The high DMFT values observed in our sample indicate a substantial cumulative burden of untreated and previously treated dental disease, suggesting delayed or insufficient access to dental care. This pattern suggests limited engagement with preventive and therapeutic dental services, likely resulting in disease progression before intervention. Similarly, the elevated OHI-S scores reflect poor oral hygiene practices and persistent plaque accumulation, which may further increase the risk of periodontal inflammation and progression of periodontal disease [27,40]. These findings may reflect a cumulative effect of biological, behavioral, and social factors on oral health deterioration in this population. OHRQoL, assessed using the OHIP-14, was notably impaired, with high total scores and the greatest impact observed in the domains of handicap, psychological discomfort, and psychological and social disability, consistent with previous findings [4,13,14,15,21]. The high prevalence of impact underscores the substantial burden of oral health–related impairment and suggests that psychological and social dimensions of oral health may be particularly affected in this population. These findings are in line with studies among individuals with substance use disorders, including people who inject drugs, which have reported similarly elevated OHIP-14 scores and extent of impact [13,14,21,41].

The regression analysis suggested that social vulnerability, greater caries experience, and poorer oral hygiene are important contributors to worse OHRQoL in this population. These findings highlight the interplay between structural and clinical determinants of oral health and indicate that management of patients with heroin-related opioid use disorder should address both oral disease and broader social circumstances. Housing instability, a well-established social determinant, may limit access to care and contribute to chronic stress and neglect of oral hygiene [25,42,43]. Greater caries experience was associated with poorer OHRQoL, indicating that the burden of dental caries is not only objectively measurable but also subjectively experienced as an important factor affecting daily functioning and overall well-being. Elevated DMFT values have also been associated with poorer self-perceived oral health among individuals with substance use disorders [28]. Poor oral hygiene emerged as a key determinant of OHRQoL, with OHI-S also showing a significant association with reduced OHRQoL in the regression model [38].

Although self-rated oral health was negatively correlated with OHIP-14 scores, it did not remain a significant predictor in the regression model, suggesting that subjective perceptions alone may not fully capture the complexity of OHRQoL determinants [30]. Similarly, the duration of heroin use was not identified as an independent predictor, which is consistent with previous research indicating that duration alone may not explain perceived oral health impact when clinical and psychosocial factors are considered simultaneously [6]. However, individuals with a longer duration of heroin use more frequently reported poor oral health and exhibited higher DMFT scores and worse oral hygiene indicators, along with a higher prevalence of bruxism. This pattern should be interpreted cautiously, as the five-year cut-off was used only for descriptive purposes and not as a clinically meaningful threshold. The observed correlation between duration of use and OHIP-14 scores suggests a cumulative negative effect on perceived quality of life.

These findings underscore the relevance of oral healthcare in addiction treatment services, with a focus on early prevention, regular dental screening, and interventions targeting both behavioral and social determinants of health. At the same time, because all participants were hospitalized and receiving supervised methadone as part of routine inpatient care, methadone exposure in this cohort was relatively uniform and short-term; the observed oral-hygiene behaviors and self-reported oral symptoms should be interpreted within this specific clinical context. Accordingly, the present study describes a distinct inpatient population with heroin-related opioid use disorder, and the findings should be interpreted with caution when extrapolating to community-dwelling heroin users.

Limitations of this study include its cross-sectional design, which prevents causal interpretations. The sample was recruited from a single treatment center and consisted exclusively of hospitalized patients receiving supervised methadone therapy, which limits the generalizability of the findings to broader populations, particularly community-dwelling heroin users and individuals outside formal treatment settings. In addition, xerostomia was assessed only as self-reported dry mouth, without objective salivary flow measurement or severity grading, while bruxism was classified as possible bruxism based solely on patient report, without instrumental confirmation or further characterization. Finally, while OHIP-14 is a validated and widely used instrument, it captures subjective perceptions that may be influenced by broader psychological and contextual factors.

## 5. Conclusions

This study highlights significant oral health disparities and reduced OHRQoL among hospitalized patients with heroin-related opioid use disorder. High DMFT values, poor oral hygiene, and housing instability were identified as key predictors of OHRQoL. These findings highlight the potential value of integrated, prevention-focused dental care within addiction treatment settings, alongside broader efforts to address underlying social determinants. Future studies are needed to determine whether such integrated approaches improve oral health outcomes in this population.

## Figures and Tables

**Table 1:** Socio-demographic profile and risk behaviors of hospitalized patients with heroin-related opioid use disorder.

Socio-Demographic Characteristics	(*n* = 97)
Sex, *n* (%)	
Male	51 (52.6)
Female	46 (47.4)
Age group	
<25 years	30 (30.9)
>25 years	67 (69.1)
Marital status, *n* (%)	
Single	67 (69.1)
Married/partner	24 (24.7)
Divorced	6 (6.2)
Education level, n (%)	
No formal education	1 (1.0)
Primary school	20 (20.6)
Secondary school	64 (66.0)
University	12 (12.4)
Employment status, *n* (%)	
Employed	39 (40.2)
Unemployed	58 (59.8)
Accommodation, *n* (%)	
Stable	61 (62.9)
Unstable	27 (27.8)
Homeless	9 (9.3)
Self-perceived economic status, *n* (%)	
Very good and good	33 (34.0)
Moderate	29 (29.9)
Bad and very bad	35 (36.1)
**Risk behavior**	
Smoking status, *n* (%)	
Smoker	94 (96.9)
Non-smoker	3 (3.1)
Smoking duration? (years)	
Non-smoker	3 (3.1)
<5 years	9 (9.3)
5–10 years	31 (32.0)
>10 years	54 (55.7)
Number of cigarettes smoked per day, *n* (%)	
Non-smoker	3 (3.1)
<20 cigarettes/day	13 (13.3)
>20 cigarettes/day	81 (83.5)
Alcohol consumption, *n* (%)	
Yes	40 (41.2)
No	57 (58.8)

**Table 2:** Oral health characteristics and OHIP-14 severity in the total sample and according to duration of heroin use (≤5 vs. >5 years) (*n* = 97).

Variable	Total Sample (*n* = 97)	≤5 Years (*n* = 35)	>5 Years (*n* = 62)	*p*-Value
**Oral health behaviors and self-reported oral symptoms**				
Brushing teeth, *n* (%)				
Does not brush	13 (13.4)	7 (20.0)	6 (9.6)	0.342
Once a day	43 (44.3)	15 (42.9)	28 (45.2)	
Two or more times a day	41 (42.3)	13 (37.1)	28 (45.2)	
When was the last time you visited the dentist? *n* (%)				
≤1 year	22 (22.7)	6 (17.1)	16 (25.8)	0.328
>1 year	75 (77.3)	29 (82.9)	46 (74.2)	
Self-assessed oral health, *n* (%)				
Very bad and bad	57 (58.8)	14 (40.0)	43 (69.4)	0.016 *
Medium	33 (34.0)	18 (51.4)	15 (24.2)	
Good and very good	7 (7.2)	3 (8.6)	4 (6.5)	
Possible bruxism, *n* (%)				
Yes	41 (42.3)	8 (22.9)	33 (53.2)	0.007 *
No	56 (57.7)	27 (77.1)	29 (46.8)	
Self-reported xerostomia, *n* (%)				
Yes	27 (27.8)	8 (22.9)	19 (30.6)	0.558
No	70 (72.2)	27 (77.1)	43 (69.4)	
**Clinical oral health indicators**				
DMFT, mean ± SD	19.1 ± 6.1	15.4 ± 6.4	21.3 ± 4.8	0.003 *
Decayed teeth (DT), mean ± SD	11.5 ± 4.9	9.6 ± 5.1	12.6 ± 4.5	<0.001 *
Missing teeth (MT), mean ± SD	5.0 ± 3.5	2.7 ± 2.1	6.2 ± 3.5	<0.001 *
Filled teeth (FT), mean ± SD	2.6 ± 2.3	3.0 ± 2.6	2.4 ± 2.0	0.418
OHI-S, mean ± SD	2.0 ± 1.2	0.8 ± 0.9	2.6 ± 0.9	<0.001 *
Debris index (DI), mean ± SD	1.6 ± 1.0	0.6 ± 0.7	2.1 ± 0.8	<0.001 *
Calculus index (CI), mean ± SD	0.4 ± 0.3	0.1 ± 0.2	0.5 ± 0.2	<0.001 *
**OHRQoL (OHIP-14 severity)**				
Total OHIP-14 score (0–56), mean ± SD	29.6 ± 7.0	26.0 ± 6.7	31.6 ± 6.4	<0.001 *
Domains (0–8)				
Functional limitation	2.2 ± 2.1	2.0 ± 1.5	2.3 ± 2.4	0.349
Physical pain	3.7 ± 2.1	2.7 ± 2.0	4.3 ± 2.0	<0.001 *
Psychological discomfort	5.3 ± 1.6	4.7 ± 1.6	5.6 ± 1.6	0.008 *
Physical disability	3.2 ± 2.3	2.3 ± 2.0	3.7 ± 2.4	0.003 *
Psychological disability	5.1 ± 1.8	4.8 ± 1.8	5.1 ± 1.9	0.304
Social disability	4.6 ± 2.0	4.2 ± 1.9	4.7 ± 2.0	0.187
Handicap	5.6 ± 1.7	5.4 ± 1.6	5.7 ± 1.7	0.337

DMFT—Decayed, Missing, and Filled Teeth; DT—Decayed Teeth; MT—Missing Teeth; FT—Filled Teeth; OHI-S—Simplified Oral Hygiene Index; DI—Debris Index; CI—Calculus Index; OHRQoL—oral health-related quality of life; OHIP-14—Oral Health Impact Profile (14-item version); SD—standard deviation; * *p* < 0.05 considered statistically significant.

**Table 3:** Correlation between OHIP-14 severity scores and selected socioeconomic, subjective, and clinical oral health parameters.

Variable	OHIP-14 Severity	
	r	*p*-Value
Self-perceived economic status	0.002	0.988
Self-assessed oral health	−0.267	0.008 *
DMFT	0.754	˂0.001 *
Decayed teeth (DT)	0.524	˂0.001 *
Missing teeth (MT)	0.489	˂0.001 *
Filled teeth (FT)	0.173	0.090
OHI-S	0.552	˂0.001 *
Debris index (DI)	0.575	˂0.001 *
Calculus index (CI)	0.301	0.003 *
Duration of heroin use	0.384	˂0.001 *

DMFT—Decayed, Missing, and Filled Teeth; DT—Decayed Teeth; MT—Missing Teeth; FT—Filled Teeth; OHI-S—Simplified Oral Hygiene Index; DI—Debris Index; CI—Calculus Index; OHIP-14—Oral Health Impact Profile (14-item version); r—Spearman’s correlation coefficient; * *p* < 0.05 considered statistically significant.

**Table 4:** Predictors of OHIP-14 severity: results of multiple linear regression analysis (*n* = 97).

Predictors	B	SE	t	β	*p*	95% CI (B)
Age	0.098	0.153	0.638	0.052	0.525	−0.208 to 0.403
Accommodation	1.754	0.683	2.567	0.166	0.012 *	0.396 to 3.111
Smoking status	0.947	2.560	0.370	0.024	0.712	−4.141 to 6.034
Self-assessed oral health	−0.025	0.547	−0.045	−0.003	0.964	−1.112 to 1.062
DMFT	0.714	0.077	9.235	0.624	<0.001 *	0.561 to 0.868
OHI-S	1.521	0.547	2.778	0.272	0.007 *	0.433 to 2.608
Duration of heroin use	0.073	1.246	0.058	0.005	0.954	−2.402 to 2.548

Dependent variable: OHIP-14—Oral Health Impact Profile (14-item version) severity (total score). B—unstandardized regression coefficient; SE—standard error; β—standardized regression coefficient; CI—confidence interval. DMFT—Decayed, Missing, and Filled Teeth; OHI-S—Simplified Oral Hygiene Index. Accommodation was coded as 0 = stable accommodation and 1 = unstable housing/homelessness; smoking status as 0 = non-smoker and 1 = smoker; self-assessed oral health was entered as an ordinal variable, with higher values indicating poorer self-perceived oral health. No missing data were observed for variables included in the regression analysis; therefore, complete-case analysis was applied, and all 97 participants were retained. No missing data were observed for variables included in the regression analysis; therefore, complete-case analysis was applied, and all 97 participants were retained. * *p* < 0.05 was considered statistically significant.

## Data Availability

Data are available upon request from the authors.

## Abbreviations

The following abbreviations are used in this manuscript:

OHRQoL	Oral health-related quality of life
WHO	World Health Organization
DMFT	Decayed, Missing, and Filled Teeth
OHIP-14	The Oral Health Impact Profile
DT	Decayed Teeth
MT	Missing Teeth
FT	Filled Teeth
OHI-S	Simplified Oral Hygiene Index
DI	Debris Index
CI	Calculus Index
